# Comparison of Different Disease-Specific Health-Related Quality of Life Measurements in Patients with Long-Term Noninvasive Ventilation

**DOI:** 10.1155/2017/8295079

**Published:** 2017-05-15

**Authors:** Toru Oga, Hiroyuki Taniguchi, Hideo Kita, Tomomasa Tsuboi, Keisuke Tomii, Morihide Ando, Eiji Kojima, Hiromi Tomioka, Yoshio Taguchi, Yusuke Kaji, Ryoji Maekura, Toru Hiraga, Naoki Sakai, Tomoki Kimura, Michiaki Mishima, Wolfram Windisch, Kazuo Chin

**Affiliations:** ^1^Department of Respiratory Care and Sleep Control Medicine, Graduate School of Medicine, Kyoto University, Kyoto, Japan; ^2^Department of Respiratory Medicine and Allergy, Tosei General Hospital, Aichi, Japan; ^3^Department of Respiratory Medicine, Takatsuki Red Cross Hospital, Osaka, Japan; ^4^Department of Respiratory Medicine, National Hospital Organization Minami-Kyoto Hospital, Kyoto, Japan; ^5^Department of Respiratory Medicine, Kobe City Medical Center General Hospital, Hyogo, Japan; ^6^Department of Pulmonary Medicine, Ogaki Municipal Hospital, Gifu, Japan; ^7^Department of Respiratory Medicine, Komaki City Hospital, Aichi, Japan; ^8^Department of Respiratory Medicine, Kobe City Medical Center West Hospital, Hyogo, Japan; ^9^Department of Respiratory Medicine, Tenri Hospital, Nara, Japan; ^10^Department of Respiratory Medicine, National Hospital Organization Toneyama Hospital, Osaka, Japan; ^11^Department of Respiratory Medicine, Otsu Red Cross Hospital, Shiga, Japan; ^12^Department of Respiratory Medicine, Graduate School of Medicine, Kyoto University, Kyoto, Japan; ^13^Department of Pneumology, Cologne Merheim Hospital, Kliniken der Stadt Köln gGmbH, Witten/Herdecke University, Faculty of Health, School of Medicine, Cologne, Germany

## Abstract

**Background:**

Two disease-specific questionnaires have been developed to assess health-related quality of life (HRQL) in patients with chronic respiratory failure: the Severe Respiratory Insufficiency (SRI) Questionnaire and the Maugeri Respiratory Failure (MRF) Questionnaire. We aimed to compare the characteristics of the SRI, MRF-26, and St. George's Respiratory Questionnaire (SGRQ) for use in patients with home noninvasive ventilation (NIV).

**Methods:**

Fifty-six outpatients receiving long-term NIV were recruited and underwent assessments of pulmonary function, arterial blood gas, HRQL, dyspnea, and psychological status.

**Results:**

Correlations of the SRI and MRF-26 with the SGRQ were modest. While pulmonary function was weakly related to only some domains of the SRI and MRF-26, the modified Medical Research Council (mMRC) dyspnea scale and Hospital Anxiety and Depression Scale (HADS) were significantly related to all domains of the SRI and MRF-26. Multiple regression analyses showed that HADS depression and mMRC accounted for 34% and 27% of the variance in the SRI, 24% and 37% in the MRF-26, and 17% and 46% in the SGRQ, respectively.

**Conclusions:**

The SRI and MRF-26 were reliable questionnaires for patients receiving long-term NIV. Dyspnea and psychological status were their main common determinants. The SRI covers more psychological health impairments than the MRF. This trial is registered with ClinicalTrials.gov Identifier: NCT00905476.

## 1. Introduction

Health-related quality of life (HRQL) is an important outcome measure in patients with chronic respiratory failure. These patients suffer physical, psychological, and social limitations in daily life due to severely progressive underlying diseases, and cure is not possible in the vast majority of patients. The St. George's Respiratory Questionnaire (SGRQ) [1] has been the most widely used respiratory-specific HRQL measurement as its use has been validated in various respiratory disorders including chronic obstructive pulmonary disease (COPD), idiopathic pulmonary fibrosis, and treated pulmonary tuberculosis [1–3]. However, it was originally designed for patients with mild to moderate chronic airway obstruction [1] and was not validated in patients with chronic respiratory failure.

Thus, for HRQL assessment of these patients, the Maugeri Respiratory Failure (MRF) Questionnaire [4] was firstly developed. Then, more specifically for patients receiving home noninvasive ventilation (NIV), the Severe Respiratory Insufficiency (SRI) Questionnaire [5] was developed and validated as a multidimensional instrument with high psychometric properties. Two previous studies [6, 7] compared these two respiratory failure-specific questionnaires with regard to their determinants together with the COPD-specific questionnaire, Chronic Respiratory Disease Questionnaire [8], and showed that they were both reliable and valid; however, these studies were limited to a homogeneous group of participants with COPD. The purpose of the present study is to investigate the relationships of the SRI, MRF, and SGRQ with clinical measurements and to clarify their characteristics in a heterogeneous group of patients with chronic respiratory failure and the need for long-term NIV.

## 2. Methods

### 2.1. Participants

We recruited 56 stable outpatients receiving NIV due to chronic hypercapnic respiratory failure as described in detail previously [9, 10]. Their primary disorders were limited to COPD and/or pulmonary tuberculosis sequelae. They had no tracheotomy and no uncontrolled comorbidities such as malignant disorders, cardiovascular diseases, or cerebrovascular diseases. We assessed their body mass index (BMI), smoking status, pulmonary function, arterial blood gas, and patient-reported measurements of HRQL, dyspnea, and psychological status. Regarding pulmonary function, forced expiratory volume in one second (FEV_1_) and forced vital capacity (FVC) were measured. All patients gave written informed consent, and the study was approved by the Ethical Committee of Kyoto University and each local facility.

### 2.2. Patient-Reported Measurements

HRQL was evaluated using the Japanese versions of the SGRQ (respiratory-specific questionnaire) [1, 11], SRI [5, 9, 10, 12] and MRF-26 [4, 10, 13] (both for respiratory failure-specific questionnaire). The SGRQ was originally developed for patients with chronic airflow limitation such as asthma or COPD and subsequently was validated for various respiratory disorders. It has 3 components: symptoms, activities, and impacts, and the total score is calculated. The SRI has 7 subscales: respiratory complaints (RC: 8 items), physical functioning (PF: 6 items), attendant symptoms and sleep (AS: 7 items), social relationships (SR: 6 items), anxiety (AX: 5 items), psychological well-being (WB: 9 items), and social functioning (SF: 8 items). One summary scale (SS) is obtained from the sum of the 7 subscales. The MRF-26 [13] is a modified version of the original MRF-28 [4] designed for patients with chronic respiratory failure. It has two domains: daily activities and perceived disability, from which the total score is calculated. In each questionnaire, the score ranges from 0 to 100: higher scores indicate a better HRQL in the SRI and vice versa in the SGRQ and MRF-26.

Dyspnea during activities of daily living was evaluated by the modified Medical Research Council (mMRC) dyspnea scale [10, 14]. mMRC is a unidimensional 5-point scale (0–4) based on degrees of various physical activities that precipitate dyspnea. Higher scores indicate worse dyspnea. Psychological status was assessed using the Japanese version of the Hospital Anxiety and Depression Scale (HADS) [10, 15], measuring anxiety and depression. Each subscale score ranges from 0 to 21, with higher scores indicating a poor psychological status.

### 2.3. Statistical Analysis

Results are expressed as mean ± standard deviation. Spearman rank correlation tests were performed to analyze the relationships between two sets of data. Stepwise multiple regression analyses were used to identify those variables that could best predict the HRQL scores using variables that were significant between two sets of data. *p* value of less than 0.05 was considered to be statistically significant.

## 3. Results

### 3.1. Patients

Baseline characteristics of 56 patients receiving long-term NIV due to chronic hypercapnic respiratory failure are shown in Table 1 and also have been reported previously [9, 10]. Their primary underlying disorders were COPD (23 patients), pulmonary tuberculosis sequelae (31 patients), and both (2 patients). These two diseases are the most common causes of the need for long-term NIV, which accounted for 26% and 23%, respectively, of the total patients receiving home NIV based on the White Paper 2010 published in Japan.

### 3.2. Concurrent Validity among HRQL Questionnaires

Spearman's rank correlation coefficients (*R*_s_) between the domain scores and total scores of the SRI, MRF-26, and SGRQ were modest but significant except for one insignificant relationship between the SRI-AS and SGRQ symptoms (Table 2). High correlations (*R*_s_ > 0.70) were found between the MRF-26 daily activities and SGRQ activities (*R*_s_ = 0.82) and impacts (*R*_s_ = 0.73).

### 3.3. Relationship between HRQL and Other Clinical Measurements

In all 3 instruments, SRI, MRF-26, and SGRQ, there was a significant relationship with mMRC dyspnea and HADS anxiety and depression (Table 3). Regarding physical domains, mMRC dyspnea had a moderate relationship with SRI-PF (*R*_s_ = 0.45) and a strong relationship with daily activities in the MRF-26 and activities in the SGRQ (*R*_s_ = 0.72). Regarding the psychological domains, the HADS had strong correlations with SRI-AX and SRI-WB (*R*_s_ = 0.51–0.67).

BMI was partly weakly but significantly related to SRI-PF, SRI-SR, SRI-SS, and daily activities and the total of the MRF-26 (*R*_s_ = 0.29–0.38). Pulmonary function was also partly significantly related to SRI-RC, SRI-WB, SRI-SS (*R*_s_ = 0.28–0.39), two domains and total of the MRF-26 (*R*_s_ = 0.35–0.49) and symptoms, activities, and total of the SGRQ (*R*_s_ = 0.31–0.42). Regarding arterial blood gas parameters, arterial carbon dioxide pressure (PaCO_2_) and base excess (BE) were weakly but significantly related with some domains of the SRI and MRF-26 but no components of the SGRQ.

Multiple regression analyses were performed to investigate factors explaining summary or total HRQL scores using 2 models (excluding or including patient-reported measurements of dyspnea and depression (Models I and II, resp.)) (Table 4). In Model I, among physiological measurements, BMI and FVC together accounted for 25% of the variance in the MRF-26, while 15% and 9% were explained by PaCO_2_ in the SRI and by FEV_1_ in the SGRQ, respectively. In Model II, mMRC dyspnea and HADS depression significantly explained 61%, 62%, and 63% of the variance in the SRI, MRF-26, and SGRQ, respectively, and BMI only explained 5% of the variance in the MRF-26. In the SRI, HADS depression most accounted for 34% of the variance, while mMRC most accounted for 37% of the variance in the MRF-26 and 46% of the variance in the SGRQ.

## 4. Discussion

We cross-sectionally evaluated and compared two respiratory failure-specific questionnaires, the SRI and MRF-26, in addition to the SGRQ. We found that both were valid in patients receiving NIV due to chronic hypercapnic respiratory failure. Dyspnea and psychological status were the major determinants in the multiple regression analyses. There were some differences in their relationships with physiological measurements.

In a recent clinical trial, disease-specific questionnaires were shown to be capable of detecting beneficial changes in HRQL following NIV treatments in patients with severe COPD apart from the generic questionnaire [16]. We then tested whether the Japanese versions of the SRI and MRF-26 yielded similar results to those of an established HRQL instrument and found that their results were mostly significantly related to the SGRQ. The SRI and MRF-26 contain items on specific problems that patients with chronic respiratory failure would encounter unlike the SGRQ. Therefore, those two highly specific questionnaires may be preferred in addition to or instead of the SGRQ in patients with chronic respiratory failure.

We also examined to what extent HRQL scores were correlated with relevant clinical measurements. The mMRC and HADS were significantly related to all domains of the SRI, MRF-26, and SGRQ. Thus, the SRI and MRF-26 reflected physical and psychological impairments in health similarly to the well-established SGRQ, although the MRF-26 had no specific psychological domains. In contrast, relationships between HRQL and physiological measurements were not strong, indicating that HRQL is somewhat independent of physiological parameters, and cannot be estimated from pulmonary function or blood gases alone. Regarding pulmonary function, FEV_1_ and FVC were significantly but weakly related to only some domains of the HRQL like SRI-RC, daily activities in the MRF-26, and activity in the SGRQ, but most relationships were nonsignificant. In addition, arterial blood gas measurements such as PaCO_2_ and BE were significantly weakly related to some domains of the SRI and MRF-26, but not the SGRQ. This may be connected to the notion that those instruments were originally developed specifically for severely affected patients with arterial blood gas abnormalities, unlike the SGRQ.

This is the first study comparing the determinants of the SRI, MRF-26, and SGRQ in patients with chronic respiratory failure receiving NIV. Multiple regression analyses showed that dyspnea and depression were their main determinants, explaining over 60% of the variance. In studies comparing the SRI and MRF-28 (the earlier version of the MRF-26) in patients with severe COPD [6, 7], the SRI was especially related to psychological status, similarly to our study, but, by entering the Groningen Activity Restriction Scale, the MRF-28 placed more emphasis on restriction in the degree of activities of daily living rather than psychological status. Although we did not assess activity limitations specifically, the items on the mMRC scale also assess such limitations in addition to dyspnea [17]: therefore, the mMRC was the most important determinant of the MRF-26 (37%) and SGRQ (46%) in contrast to the SRI (27%).

In multiple regression analyses, where explanatory factors were limited to BMI, pulmonary function, and arterial blood gas, the MRF-26 was explained by 25% of the variance, which was more than in the SRI (15%) and SGRQ (9%). This indicates that the MRF-26 placed greater emphasis on daily restrictions due to physiological impairments than the SRI. In a longitudinal analysis in patients receiving NIV, although both the SRI and MRF-26 strongly predicted mortality in univariate analyses, the SRI was predictive independently of BMI, FVC, and PaCO_2_, but the MRF-26 was not [10]. This may be related to the possible relationship between MRF-26 and physiological measurements.

The SRI contains 49 items divided into 7 subscales, and each is scored by a 5-point Likert scale while the MRF-26 contains 26 items divided into only 2 domains, and each is measured with yes/no. Thus, structurally, the SRI would measure diversified health impairments more discriminatively than the MRF-26, although it may take more time to be administered. While the MRF was developed for patients with chronic respiratory failure irrespective of NIV, the SRI was primarily developed for patients with home NIV, and it recently was validated in patients with COPD receiving long-term oxygen therapy [18]. As a limitation of the present study, we did not compare responsiveness of the questionnaires, and this should be studied in the near future. Thus, considering various factors is important when choosing questionnaires.

As another limitation of the present study, the sample size was relatively small. Unfortunately, we could not collect a larger number of such severely ill patients who needed long-term NIV. However, we consider that an increase in the sample size would not greatly change the main results regarding the characteristics of the questionnaires assessed by their relationships with other clinical measures.

We demonstrated that both the SRI and MRF-26 were reliable questionnaires in patients with chronic hypercapnic respiratory failure receiving NIV. Dyspnea and psychological status were similarly their main determinants while their relationships with physiological measurements were weaker. The SRI covers more psychological health impairments than the MRF.

## Figures and Tables

**Table 1:** Baseline characteristics of 56 patients using noninvasive ventilation.

Characteristics	Total (*n* = 56)	COPD (*n* = 23)	Tb (*n* = 31)
Age, years	73 ± 8	70 ± 8	76 ± 7
BMI, kg/m^2^	20.5 ± 3.7	20.6 ± 3.8	20.4 ± 3.8
Cumulative smoking, pack-years	29 ± 34	50 ± 31	12 ± 26
FEV_1_, % predicted	29.9 ± 10.6	25.7 ± 10.3	33.0 ± 10.3
FVC, % predicted	45.7 ± 18.7	56.1 ± 22.7	38.1 ± 11.0
PaO_2_, kPa	10.7 ± 2.2	10.2 ± 2.2	11.1 ± 2.2
PaCO_2_, kPa	7.7 ± 1.4	7.5 ± 1.5	7.9 ± 1.4
Base excess, mmol/L	5.4 ± 3.4	4.8 ± 3.0	6.0 ± 3.6
mMRC dyspnea	2.3 ± 1.1	2.3 ± 1.0	2.3 ± 1.1
HADS anxiety	5.6 ± 3.7	5.5 ± 3.9	5.7 ± 3.6
HADS depression	7.4 ± 3.8	7.3 ± 3.5	7.5 ± 4.0
SRI respiratory complaints	62.3 ± 21.1	61.0 ± 21.2	62.5 ± 21.5
SRI physical functioning	52.1 ± 19.2	55.3 ± 17.3	50.5 ± 20.7
SRI attendant symptoms and sleep	61.3 ± 16.8	58.4 ± 18.1	63.0 ± 16.0
SRI social relationships	53.5 ± 17.6	55.6 ± 15.4	51.8 ± 20.0
SRI anxiety	54.2 ± 24.3	55.0 ± 24.0	52.7 ± 25.4
SRI psychological well-being	54.3 ± 17.0	55.1 ± 18.0	53.8 ± 17.0
SRI social functioning	54.5 ± 21.1	56.1 ± 18.9	54.0 ± 23.3
SRI summary	56.0 ± 15.3	56.6 ± 14.7	55.5 ± 16.4
MRF-26 daily activities	43.2 ± 30.5	39.8 ± 28.4	44.9 ± 31.9
MRF-26 perceived disability	52.7 ± 28.3	48.4 ± 26.9	54.3 ± 29.5
MRF-26 total	48.0 ± 26.7	43.8 ± 22.8	49.9 ± 29.1
SGRQ symptoms	50.4 ± 22.1	50.9 ± 23.6	49.6 ± 22.1
SGRQ activities	74.1 ± 17.4	75.7 ± 16.4	72.8 ± 18.5
SGRQ impacts	44.6 ± 22.4	45.3 ± 23.4	43.0 ± 22.2
SGRQ total	54.5 ± 18.5	55.4 ± 19.1	53.1 ± 18.5

Theoretical scores range from 0 to 4 in the mMRC, 0 to 21 in the HADS, and 0 to 100 in the SRI, MRF-26, and SGRQ. For the SRI, minimum scores indicate poor status, and for the mMRC, HADS, MRF-26, and SGRQ, maximum scores indicate poor status; BMI = body mass index; COPD = chronic obstructive pulmonary disease; Tb = pulmonary tuberculosis sequelae; FEV_1_ = forced expiratory volume in one second; FVC = forced vital capacity; PaO_2_ = arterial oxygen pressure; PaCO_2_ = arterial carbon dioxide pressure; mMRC = modified Medical Research Council; HADS = Hospital Anxiety and Depression Scale; SRI = Severe Respiratory Insufficiency; MRF = Maugeri Respiratory Failure; SGRQ = St. George's Respiratory Questionnaire.

**Table 2:** Correlations between HRQL questionnaires.

	SGRQ			
	Symptoms	Activities	Impacts	Total
SRI				
Respiratory complaints	−0.61	−0.69	−0.69	−0.74
Physical functioning	−0.32	−0.52	−0.48	−0.50
Attendant symptoms and sleep	—	−0.34	−0.33	−0.35
Social relationships	−0.40	−0.34	−0.49	−0.48
Anxiety	−0.36	−0.30	−0.49	−0.48
Psychological well-being	−0.44	−0.34	−0.55	−0.54
Social functioning	−0.45	−0.46	−0.66	−0.65
Summary score	−0.54	−0.55	−0.69	−0.71
MRF-26				
Daily activities	0.50	**0.82**	**0.73**	0.80
Perceived disability	0.46	0.48	0.69	0.69
Total	0.51	0.73	0.78	0.81

Missing values (—) indicate no significant relationships (*p* > 0.05). High correlations (correlation coefficient > 0.70), excluding summary and total scores, are in bold; HRQL = health-related quality of life; SRI = Severe Respiratory Insufficiency; MRF = Maugeri Respiratory Failure; SGRQ = St. George's Respiratory Questionnaire.

**Table 3:** Correlations between HRQL questionnaires and physiological measures.

	SRI								MRF-26			SGRQ			
	RC	PF	AS	SR	AX	WB	SF	SS	Dai	Per	Tot	Sym	Act	Imp	Tot
Age	—	—	—	—	—	—	—	—	—	—	—	—	—	—	—
BMI	—	0.31	—	0.31	—	—	—	0.29	−0.38	—	−0.33	—	—	—	—
Smoking	—	—	—	—	—	—	—	—	—	—	—	—	—	—	—
FEV_1_	0.30	—	—	—	—	—	—	—	−0.35	—	—	−0.31	−0.40	—	−0.31
FVC	0.39	—	—	—	—	0.28	—	0.30	−0.49	−0.38	−0.47	—	−0.42	—	−0.33
PaO_2_	—	—	—	—	—	—	—	—	—	—	—	—	—	—	—
PaCO_2_	−0.41	—	—	−0.28	—	—	−0.38	−0.38	0.38	—	0.33	—	—	—	—
BE	—	—	—	—	—	—	−0.30	−0.28	0.34	—	0.27	—	—	—	—
Dyspnea	−0.60	−0.45	−0.29	−0.46	−0.31	−0.42	−0.45	−0.56	0.72	0.46	0.51	0.59	0.72	0.62	0.69
Anxiety	−0.60	−0.34	−0.54	−0.50	−0.51	−0.67	−0.68	−0.69	0.46	0.57	0.73	0.39	0.36	0.51	0.51
Depression	−0.60	−0.48	−0.50	−0.44	−0.60	−0.56	−0.53	−0.70	0.58	0.62	0.78	0.37	0.45	0.59	0.59

Missing values (—) indicate no significant relationships (*p* > 0.05). SRI, Severe Respiratory Insufficiency; RC, respiratory complaints; PF, physical functioning; AS, attendant symptoms and sleep; SR, social relationships; AX, anxiety; WB, psychological well-being; SF, social functioning; SS, summary score; MRF, Maugeri Respiratory Failure; Dai, daily activities; Per, perceived disability; Tot, total; SGRQ, St. George's Respiratory Questionnaire; Sym, symptoms; Act, activities; Imp, impacts; BMI, body mass index; FEV_1_, forced expiratory volume in one second; FVC, forced vital capacity; PaO_2_, arterial oxygen pressure; PaCO_2_, arterial carbon dioxide pressure; BE, base excess.

**Table 4:** Results of multiple regression analyses.

	SRI summary		MRF-26 total		SGRQ total	
	*β*	*R* ^2^	*β*	*R* ^2^	*β*	*R* ^2^
*Model I (excluding patient-reported measurements)*						
BMI, kg/m^2^	—	—	−0.27	0.09		
FEV_1_, % predicted					−0.31	0.09
FVC, % predicted	—	—	−0.38	0.16	—	—
PaCO_2_, kPa	−0.31	0.15	—	—		
BE, mmol/L	—	—	—	—		

Cumulative *R*^2^		0.15		0.25		0.09

*Model II (including patient-reported measurements)*						
BMI, kg/m^2^	—	—	−0.17	0.05		
FEV_1_, % predicted					—	—
FVC, % predicted	—	—	—	—	—	—
PaCO_2_, kPa	—	—	—	—		
BE, mmol/L	—	—	—	—		
mMRC dyspnea	−0.45	0.27	0.55	0.37	0.63	0.46
HADS depression	−0.49	0.34	0.37	0.24	0.29	0.17

Cumulative *R*^2^		0.61		0.67		0.63

All values represent coefficient of determination (*R*^2^). Missing values (—) indicate no significant relationships; SRI = Severe Respiratory Insufficiency; MRF = Maugeri Respiratory Failure; SGRQ = St. George's Respiratory Questionnaire; BMI = body mass index; FEV_1_ = forced expiratory volume in one second; FVC = forced vital capacity; PaO_2_ = arterial oxygen pressure; PaCO_2_ = arterial carbon dioxide pressure; BE = base excess; mMRC = modified Medical Research Council; HADS = Hospital Anxiety and Depression Scale.
